# Identity of *Fasciola* spp. in sheep in Egypt

**DOI:** 10.1186/s13071-016-1898-2

**Published:** 2016-12-01

**Authors:** Said Amer, Ahmed ElKhatam, Shereif Zidan, Yaoyu Feng, Lihua Xiao

**Affiliations:** 1 Division of Foodborne, Waterborne and Environmental Diseases, National Center for Emerging and Zoonotic Infectious Diseases, Centers for Disease Control and Prevention, 1600 Clifton Rd, Atlanta, GA USA; 2 Department of Zoology, Faculty of Science, Kafr El sheikh University, Kafr El Sheikh, Egypt; 3 Department of Parasitology, Faculty of Veterinary Medicine, University of Sadat City, Menofia, Egypt; 4 Department of Animal Hygiene and Zoonoses, Faculty of Veterinary Medicine, University of Sadat City, Menofia, Egypt; 5 College of Veterinary Medicine, South China Agricultural University, Guangzhou, People’s Republic of China

**Keywords:** *Fasciola*, Genotype, ITS1, *nad*1, Hybridization, Egypt

## Abstract

**Background:**

In Egypt, liver flukes, *Fasciola* spp. (Digenea: Fasciolidae), have a serious impact on the farming industry and public health. Both *Fasciola hepatica* and *Fasciola gigantica* are known to occur in cattle, providing the opportunity for genetic recombination. Little is known on the identity and genetic variability of *Fasciola* populations in sheep.

**Methods:**

This study was performed to determine the prevalence of liver flukes in sheep in Menofia Province as a representative area of the delta region in Egypt, as measured by postmortem examination of slaughtered animals at three abattoirs. The identity and genetic variability of *Fasciola* spp. in slaughtered animals were determined by PCR-sequence analysis of the nuclear ribosomal internal transcribed spacer 1 (ITS1) and the mitochondrial NADH dehydrogenase subunit 1 (*nad*1) genes.

**Results:**

Physical inspection of the liver indicated that 302 of 2058 (14.7%) slaughtered sheep were infected with *Fasciola* spp. Sequence analysis of the ITS1 and *nad*1 genes of liver flukes from 17 animals revealed that 11 animals were infected with *F. hepatica*, four with *F. gigantica*, and two with both species. Seventy eight of 103 flukes genetically characterized from these animals were *F. hepatica*, 23 were *F. gigantica,* and two had ITS1 sequences identical to *F. hepatica* but *nad*1 sequences identical to *F. gigantica. nad*1 sequences of Egyptian isolates of *F. gigantica* showed pronounced differences from those in the GenBank database. Egyptian *F. gigantica* haplotypes formed haplogroup D, which clustered in a sister clade with haplogroups A, B and C circulating in Asia, indicating the existence of geographic isolation in the species.

**Conclusions:**

Both *F. hepatica* and *F. gigantica* are prevalent in sheep in Egypt and an introgressed form of the two occurs as the result of genetic recombination. In addition, a geographically isolated *F. gigantica* population is present in the country. The importance of these observations in epidemiology of fascioliasis needs to be examined in future studies.

## Background

Fascioliasis is a foodborne disease caused by infection with liver flukes of the genus *Fasciola* and occurs in a wide range of mammalian hosts worldwide [1, 2]. Liver flukes reside in the bile duct of the definitive hosts, resulting in sever hepatic damage and associated health consequences [3]. In developing countries such as African nations, *Fasciola* infections have been recognized as a major constraint to animal farming [1, 4], contributing to the impeded economic development [5]. In addition, the incidence rate of human fascioliasis is high in areas of high animal infections [6]. The World Health Organization estimates that at least 2.4 million people in more than 70 countries are affected by fascioliasis (http://www.who.int/foodborne_trematode_infections/fascioliasis/en/). Being a multifactorial disease with limited drugs available for treatment, effective measures are urgently needed to control this important foodborne and zoonotic disease [7].

*Fasciola hepatica* (Linnaeus, 1758) and *Fasciola gigantica* (Cobbold, 1856) are the causative agents of the disease in both humans and animals. The distribution of the two species of *Fasciola* appears to be geographically associated. *F. hepatica* is common in temperate zones especially Europe, Americas and Australia, while *F. gigantica* is the known species in tropical regions of Africa and Asia. Both species overlap in occurrence in subtropical areas [1, 2, 8–11]. *Fasciola* spp. have  the ability to self-fertilize, cross-fertilize and in some cases undergo parthenogenesis [12]. Hybridization between the two *Fasciola* species has been documented, leading to the emergence of intermediate forms with mixed phenotypic characteristics and genetic structure [13–15]. *Fasciola* flukes of abnormal ploidy (triploid and mixoploid) have been reported; they are parthenogenetic with no evidence of sperm production [14, 16, 17].

Molecular analyses play a pivotal role in the identification of *Fasciola* spp. [18–20], resolving morphometric discrepancies associated with species identifications, especially those related to intermediate forms. Molecular analyses of the intermediate forms have detected individuals that have divergent copies of the nuclear ribosomal genes derived from both *Fasciola* species (the hybrid form), as well as individuals with nuclear DNA of one species while mitochondrial DNA of the other species (the introgressed form) [8, 14, 17, 21]. Noteworthy, all analyzed *Fasciola* flukes in Japan and South Korea are aspermic [21–23] and have mixed *F. hepatica* and *F. gigantica* sequences by analysis of the nuclear phosphoenolpyruvate carboxykinase and DNA polymerase delta genes, suggesting that the flukes are descendants of hybridization between the two species [24].

Egypt is one of the fascioliasis-endemic areas in the world [25]. The disease burden is high in several species of livestock [26] as well as humans [27]. Both species of *Fasciola* are present in cattle in Egypt, and the occurrence of the hybrid form has been reported [8]. Thus far, the introgressed form of *Fasciola* spp. has not been detected.

In contrast to cattle, no studies are available on the molecular identity of *Fasciola* spp. in sheep in Egypt. The present study was conducted to determine the occurrence rate of *Fasciola* spp. in sheep as measured by postmortem examination of slaughtered animals at abattoirs. In addition, the identity and genetic variability of *Fasciola* spp. derived from slaughtered animals were examined by PCR-sequence analysis of the nuclear ribosomal internal transcribed spacer 1 (ITS1) and the mitochondrial NADH dehydrogenase subunit 1 (*nad*1) gene.

## Methods

### Specimen collection

Livers from 2058 slaughtered adult sheep were collected during August 2012 to August 2014 during post-mortem inspection by veterinary officers at Shebein El Kom, Ashmoun and El Shouhada abattoirs in El Menofia Province (90 km East of Cairo), Egypt. The inspected sheep included 783 animals at Shebein El Kom, 1219 at Ashmoun, and 56 at El Shouhada abattoirs. The livers were physically inspected for the presence of *Fasciola* worms. Flukes from each infected individual were collected in plastic containers, washed in physiological saline and fixed in 95% ethanol. A total of 5–7 worms from 17 infected individuals were used in molecular analysis, resulting in 103 worms genetically characterized. Randomly selected individuals (11 of each *Fasciola* species) from genetically characterized worms (i.e. hologenophore specimens according to Astrin et al. [28]) were lightly pressed between two glass slides and used in morphometric analysis.

### Morphometric analysis

Individual flukes were washed three times in PBS, stained in acetocarmine, and mounted in DPX medium [29]. Measurements, expressed in millimeters (mm) were made for 11 flukes of each *Fasciola* species, using a microscope equipped with a calibrated ocular micrometer (Leica Microsystems GmbH, Wetzlar, Germany). Six ratios were also calculated for each *Fasciola* type. Statistical analysis was conducted using the Student’s *t*-test implemented in SPSS 15.0 (SPSS, Chicago, Illinois), with values of *P* ≤ 0.05 at degree of freedom 20 considered significant.

**Table 1:** Details of *Fasciola nad*1 sequences from Egypt and other countries used in phylogenetic analysis

Accession number	Species	Host	Location	Reference
LC076235 (4 replicates)	*F. hepatica*	Sheep	Egypt	This study
LC076228 (10 replicates)	*F. hepatica*	Sheep	Egypt	This study
LC076258 (3 replicates)	*F. hepatica*	Cattle	Egypt	This study
LC076241 (4 replicates)	*F. hepatica*	Sheep	Egypt	This study
LC076240 (6 replicates)	*F. hepatica*	Sheep	Egypt	This study
LC076271 (51 replicates)	*F. hepatica*	Sheep	Egypt	This study
AB554188	*F. hepatica*	Sheep	Egypt	[8]
LC070666	*F. hepatica*	Cattle	Peru	[11]
KF111630	*F. hepatica*	Sheep	Spain	[49]
KF111634	*F. hepatica*	Sheep	Spain	[49]
KF111652	*F. hepatica*	Sheep	Spain	[​^49^]
KF111640	*F. hepatica*	Sheep	Spain	[​^49^]
AB207156	*F. hepatica*	Cattle	Ireland	[21]
AB554179	*F. hepatica*	Sheep	Egypt	[8]
AB554180	*F. hepatica*	Sheep	Egypt	[8]
AB554190	*F. hepatica*	Buffalo	Egypt	[8]
AB477359	*F. hepatica*	Cattle	China	[50]
AB554185	*F. hepatica*	Sheep	Egypt	[8]
AB554181	*F. hepatica*	Sheep	Egypt	[8]
AB554183	*F. hepatica*	Sheep	Egypt	[8]
AB554186	*F. hepatica*	Sheep	Egypt	[8]
AB554194	*Fasciola* sp.	Buffalo	Egypt	[8]
LC076204 (3 replicates)	*F. gigantica*	Sheep	Egypt	This study
LC076199 (6 replicates)	*F. gigantica*	Sheep	Egypt	This study
LC076218 (16 replicates)	*F. gigantica*	Sheep	Egypt	This study
AB554162	*F. gigantica*	Cattle	Egypt	[8]
AB554167	*F. gigantica*	Cattle	Egypt	[8]
AB554165	*F. gigantica*	Cattle	Egypt	[8]
AB554154	*F. gigantica*	Buffalo	Egypt	[8]
AB554156	*F. gigantica*	Buffalo	Egypt	[8]
LC012900	*F. gigantica*	Cattle	India	[20]
LC128314	*F. gigantica*	Buffalo	India	[53]
AB894337	*F. gigantica*	Buffalo	Nepal	[52]
AB894370	*F. gigantica*	Capra	Bangladesh	[57]
AB604007	*F. gigantica*	Ruminant	Myanmar	[51]
LC012899	*F. gigantica*	Cattle	India	[20]
LC012897	*F. gigantica*	Cattle	India	[20]
LC127275	*F. gigantica*	Ruminant	Indonesia	[54]
LC127277	*F. gigantica*	Ruminant	Indonesia	[54]
LC127264	*F. gigantica*	Ruminant	Indonesia	[54]
AB385616	*F. gigantica*	Cattle	Vietnam	[17]
AB603724	*F. gigantica*	Cattle	Thailand	[44]
AB983822	*F. gigantica*	Cattle	Zambia	^a^
AB983824	*F. gigantica*	Cattle	Zambia	^a^
AB983823	*F. gigantica*	Cattle	Zambia	^a^
AF219379	*P. westermani*			^a^

^a^GenBank (unpublished data)

### DNA extraction and PCR analysis

Individual flukes fixed in ethanol were washed extensively with PBS. To avoid the inclusion of female genitalia that might contain foreign sperms, genomic DNA was extracted from a small portion of lateral margin of the posterior end using the FastDNA SPIN Kit for Soil (MP Biomedicals, Santa Ana, CA, USA). Some of the genetically characterized flukes were used in morphometric measurement described above. The complete nuclear ITS1 and partial mitochondrial *nad*1 genes in the extracted DNA were amplified using primers of Itagaki et al. [21]. PCR reactions were done in 50 μl volume consisted of 1 μl of genomic DNA, 5 μl 10× GeneAmp PCR buffer (Applied Biosystems, Foster City, CA, USA), 8 μl of dNTP (Promega, Madison, WI, USA), 3 μl MgCl_2_, 1.5 μl of each primer, 0.3 μl of *GoTaq* DNA polymerase (Promega) and 29.7 μl of molecular grade H_2_O. Each PCR consisted of 30 cycles of denaturation at 98 °C for 10 s, annealing at 56 °C (for ITS1) or 53 °C (for *nad*1) for 35 s, and extension at 68 °C for 50 s, with an initial denaturation step at 95 °C for 5 min and a final extension step at 68 °C for 10 min. PCR products were visualized by electrophoresis in 1.5% agarose gels.

### DNA sequence analysis

PCR products were sequenced directly using the Big Dye® Terminator v3.1 Cycle Sequencing Kit and an ABI 3130 Genetic Analyzer (Applied Biosystems, Foster City, CA, USA). Sequences obtained were assembled using the ChromasPro (version 1.5) software (http://www.technelysium.com.au/ChromasPro.html). The accuracy of data was confirmed by bi-directional sequencing. The obtained sequences from each genetic target were aligned with each other and reference sequences using ClustalX (http://www.clustal.org/) to determine the identity of *Fasciola* spp. Evolutionary relationship was inferred based on *nad*1 sequences using the Maximum Likelihood (ML) method implemented in MEGA7 (http://www.megasoftware.net/). The ML phylogenetic analysis was conducted using the Kimura 2-parameter model with 1000 bootstrap replicates. A nucleotide sequence from *Paragonimus westermani* (AF219379) was used as the outgroup to root the tree. Details of sequences from Egypt and other countries used in the construction of the phylogenetic tree are shown in Table 1. Additional phylogenetic analyses were conducted using the Maximum Parsimony implemented in MEGA7 and Bayesian Method implemented in MrBayes (http://mrbayes.sourceforge.net/).

**Table 2:** Morphometric measurements (*n* = 11 for each species) of *Fasciola hepatica* and *Fasciola gigantica* from sheep in Egypt. Data are presented as the range followed by the mean in parentheses

Parameter	*Fasciola hepatica*	*Fasciola gigantica*	*t-*value	*P-*value (2-tailed)
Total length	29.60–20.80 (25.34)	34.80–26.80 (29.9)	-4.18	0.0001*
Maximum width	10.80–7.20 (9.65)	10.80–7.80 (8.75)	2.44	0.0242*
Shoulder breadth	7.60–4.80 (5.80)	6.00–4.00 (5.42)	1.14	0.2671
Oral cone length	3.60–2.00 (2.85)	3.20–2.20 (2.73)	0.67	0.5102
Oral sucker (L × W)	0.63–0.40 × 1.08–0.60 (0.53 × 0.76)	0.64–0.40 × 0.92–0.60 (0.51 × 0.79)	-0.65	0.5253
Ventral sucker (L × W)	1.20–0.80 × 1.32–0.92 (1.09 × 1.18)	1.32–0.88 × 1.32–1.00 (1.12 × 1.21)	-0.65	0.5211
Pharynx length	0.80–0.60 (0.65)	0.80–0.60 (0.65)	0.23	0.8173
Oesophagus length	1.20–1.00 (1.10)	1.28–0.80 (1.09)	0.15	0.8842
Maximum width/Total length	0.36–0.34 (0.38)	0.31–0.29 (0.29)	6.26	0.0001*
Pharynx length/Total length	0.03–0.03 (0.03)	0.02–0.02 (0.02)	3.36	0.0032*
Pharynx length/Oral cone length	0.22–0.3 (0.23)	0.25–0.27 (0.24)	-0.47	0.6443
Oesophagus length/Total length	0.04–0.05 (0.04)	0.04–0.03 (0.04)	3.41	0.0031*
Oesophagus length/Oral cone length	0.33–0.50 (0.39)	0.40–0.36 (0.40)	-0.40	0.6962
Pharynx length /Oesophagus length	0.67–0.6 (0.59)	0.63–0.75 (0.60)	-0.13	0.8981

*Abbreviations*: *L* length, *W* width

*P ≤ 0.05 (significant differences revealed by Student’s *t-*test)

Representative sequences from this study were deposited in the GenBank database under accession numbers LC076108–LC076196 for ITS1 and LC076197–LC076285 for *nad*1.

## Results

### Occurrence of liver flukes

Postmortem examinations of slaughtered sheep indicated that 302 of 2058 (14.7%) animals had *Fasciola* flukes in the bile duct. Among the three abattoirs, *Fasciola* infection rates were 9.8% (188 of 783) at Shebein El Kom, 17.8% (217 of 1219) at Ashmoun, and 14.8% (8 of 56) at El Shouhada.

### *Fasciola* species at nuclear ITS1 locus

Sequences of ~ 639 bp containing the complete ITS1 sequence and partial 18S and 5.8S rRNA gene  sequences were generated from 103 Egyptian flukes. Alignment of the sequences obtained showed the presence of 6 polymorphic sites, indicative the presence of both *F. hepatica* and *F. gigantica*. There were no nucleotide deletions between the two *Fasciola* species and, upon close examinations of the trace files from the sequencing reactions, there were no mixed peaks at any of the 6 polymorphic sites. No intra-species sequence diversity was observed at this genetic locus.

### *Fasciola* species and haplotypes at mitochondrial *nad*1 locus

Sequence analysis of the mitochondrial *nad*1 gene showed considerable genetic diversity in the form of single nucleotide polymorphism (SNPs), resulting in the identification of multiple haplotypes. A total of 42 variable sites (leading to 30 amino acid changes) representing 19 haplotypes were detected in *F. hepatica*, compared to 9 variable sites (leading to 6 amino acid changes) representing 7 haplotypes in *F. gigantica*.

**Fig. 1:**
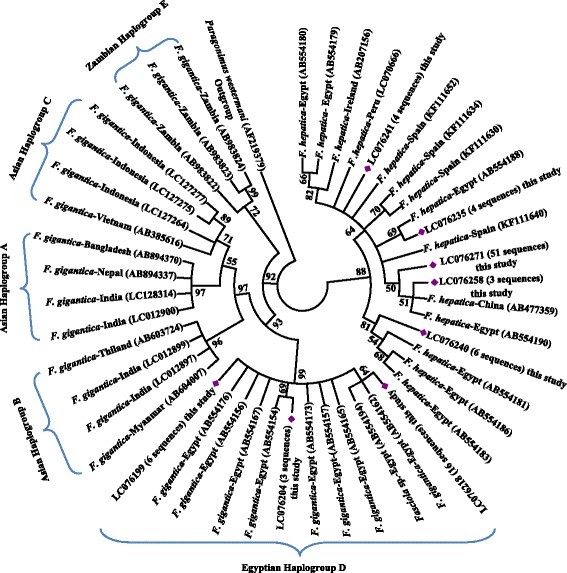
Phylogenetic relationships of *Fasciola* spp. from Egypt compared to reference *nad*1 sequences in the GenBank database based on the Maximum Likelihood analysis. Sequences obtained in this study are marked with a red diamond (see Table 1 for details on sequences used in the tree construction)

In a ML analysis of the *nad*1 sequences, all *F. gigantica* haplotypes from this study clustered with those derived from several hosts from Egypt, forming a distinct haplogroup (designated as haplogroup D). This haplogroup clustered in a sister clade with other haplogroups (A, B and C) in Asia and the haplogroup in Zambia (designated as haplogroup E) with high bootstrap value (> 90) (Fig. 1). There were about 96–99% similarities in *nad*1 sequences of *F. gigantica* between Egypt and other countries. In contrast, there were no geographic or host segregation in *F. hepatica,* as *nad*1 sequences from this study were distributed in several clusters across the tree (Fig. 1). Similar tree structures were obtained in the Maximum Parsimony and Bayesian analyses of these sequences (data not shown).

### Distribution of *Fasciola* species

Results of ITS1 and *nad*1 sequence analyses showed that 11 of the 17 fascioliasis cases characterized genetically were infections of *F. hepatica*, 4 were of *F. gigantica*, and 2 had mixed infection of both *Fasciola* species. At the individual worm level, 78 of the 103 fluke sequenced were *F. hepatica* and 23 were *F. gigantica.* The remaining two worms (Worm IDs 38314 and 38318) derived from the same sheep showed ITS1 sequences identical to *F. hepatica* and mitochondrial *nad*1 sequences identical to *F. gigantica*, representing an introgressed form of the two species.

### Morphometric characteristics

Morphometric measurements showed that *F. gigantica* was significantly longer and narrower than *F. hepatica*. Similarly, the ratios Maximum body width/Total length, Pharynx length/Total length and Oesophagus length/Total length differed significantly between the two fluke species (Table 2).

## Discussion

Sheep and goat farming is a key element in sustained economic development in developing countries [30]. Fascioliasis is a serious challenge to small ruminant farming worldwide because of the high occurrence of infection and the associated morbidity and mortality. A temporal increase in the incidence of *Fasciola* infection in sheep has been recorded over the last few decades [31, 32], and has been linked to global climate changes [33] and changes in irrigation systems, favoring the life-cycle of lymnaeid vectors. The present study showed an occurrence of *Fasciola* worms in 14.7% of sheep examined at three abattoirs in Egypt. This is lower than infection rates of 30–40% previously reported in sheep in Egypt by stool examinations [26, 34]. Prevalence rates among studies can be affected by diagnostic techniques used, age of the animals examined, time and location of the investigation [35]. Elsewhere in Africa, *Fasciola* spp. were found in 10.8% of slaughtered sheep in Algeria [36] and 23% of slaughtered sheep in Chad [37]. Similarly, *F. hepatica* and *F. gigantica* were reported in 20–26% of slaughtered sheep in Ethiopia [38].

Results reported in the present study indicate that morphometric measurements differed significantly between *F. hepatica* and *F. gigantica* in five indices including total body length, maximum width, as well as ratios Maximum body width/Total length, Pharynx length/Total length and Oesophagus length/Total length. Comparable results were previously reported on *Fasciola* flukes from different hosts [39–41]. Traditional microscopic measurements are simple and may be helpful in morphometric characterization of Fasciolids [39]. Therefore, this technique is a valuable tool in discriminating the two common *Fasciola* species in areas with low occurrence or no recorded intermediate forms, including Egypt and other African countries. However, in countries such as Japan, Vietnam and Korea, liver flukes cannot be classified as *F. hepatica* or *F. gigantica* using morphometrics because of the presence of a variety of intermediate forms [21, 22].

Molecular characterizations have identified a higher occurrence of *F. hepatica* (11/17) than *F. gigantica* (4/17), with mixed infections of both species in two slaughtered sheep. The higher prevalence of *F. hepatica* was reported by Moghaddam et al. in Iran [42], and mixed infections of the two species were previously reported in cattle in Egypt [8]. Elsewhere in Africa, *F. hepatica* is the dominant species (64%) in cattle in South Africa, although *F. gigantica* (99%) is more commonly seen in cattle in neighboring Zimbabwe [43]. In Asia, flukes recovered from cattle in Thailand were exclusively *F. gigantica* in one study [44], although Bui et al. [35] concluded that sheep in Vietnam are more susceptible to *F. hepatica* than to *F. gigantica*. The distribution of *Fasciola* species could be affected by environmental and host-related factors.

Results of the present study have shown a higher haplotype diversity of the mitochondrial *nad*1 gene in *F. hepatica* than in *F. gigantica*. This is largely in agreement with previous reports on genetic variability in mitochondrial genes *nad*1 and cytochrome oxidase 1 of *F. hepatica* by other researchers [45–50]. Cwiklinski et al. [12] concluded that the *F. hepatica* genome is highly heterogeneous. In contrast, in the previous report on bovine *Fasciola* spp. collected from Cairo, Egypt, *F. gigantica* was shown to have higher genetic diversity than *F. hepatica* [8]. Such a discrepancy between studies might be attributed to differences in the number of flukes of both species characterized.

There are apparent genetic differences in mitochondrial sequences of *F. gigantica* between Egypt and other countries. Phylogenetic analysis of *nad*1 sequences suggests that haplotypes of *F. gigantica* found in Asian countries can be categorized into three haplogroups: A, B and C [20, 44, 51, 52]. Haplogroup A is found in Indian subcontinent, including Nepal and Bangladesh [20, 52], while all three haplotypes are found in Southeast Asia, including Thailand and Myanmar [44, 51, 53, 54]. Our analysis suggested that Egyptian haplotypes of *F. gigantica* formed a monophyletic clade sister to those from Asia to the exclusion of Zambian samples (Fig. 1). In agreement with this, Ai et al. [55] described the separation of Chinese haplotypes from Niger ones.

In the present study, we identified two flukes with nuclear ITS1 data matching those of *F. hepatica* and mitochondrial *nad*1 data matching those of *F. gigantica.* This might have been caused by the occurrence introgression. Hybrid forms between *F. hepatica* and *F. gigantica* have been reported in several Asian countries [21, 22, 56, 57] and Egypt [8]. Le et al. [14] and Blair [58] defined the hybrid form as the F1 offspring of a mating between the two *Fasciola* species, carrying mitochondrial genome of the maternal parent and nuclear rRNA genes of both parents. In contrast, the introgressed form is the offspring of the back-crossing of hybrids with one parent species, which homogenizes the ribosomal array to one species and mitochondrial genome to other species (paternal introgression) or both ribosomal and mitochondrial arrays (maternal introgression) to the same species. The fact that the parasite can survive for many years in the definitive host and both *Fasciola* species have high infection rates in ruminants in some areas has apparently facilitated the occurrence of hybrid and introgressed forms. Hybrid and/or introgressed forms might play an important role in genetic diversity of *Fasciola* spp. [59], leading to potential emergence of more virulent forms. The existence of these two recombinant forms in Egypt needs confirmation using the newly developed genotyping tool targeting nuclear phosphoenolpyruvate carboxykinase and DNA polymerase delta genes [24].

## Conclusions

The present study revealed a common occurrence of *F. hepatica* and *F. gigantica* in sheep in the middle delta region of Egypt, the existence of an introgressed form of the two species in some animals, and genetic differences in *F. gigantica* between Egypt and other areas. This and the previous identification of the hybrid form of *F. hepatica* and *F. gigantica* indicate that genetic recombination may play a significant role in shaping the population structure of *Fasciola* spp. in areas with high prevalence of both *Fasciola* spp. This and its epidemiologic implications warrant future studies.

## Abbreviations

ITS1: Internal transcribed spacer 1
*nad*1: NADH dehydrogenase subunit 1
PCR: Polymerase chain reaction
